# Influence of pH and carbon to nitrogen ratio on mycotoxin production by* Alternaria alternata* in submerged cultivation

**DOI:** 10.1186/2191-0855-2-28

**Published:** 2012-05-20

**Authors:** Katrin Brzonkalik, Dominik Hümmer, Christoph Syldatk, Anke Neumann

**Affiliations:** 1 Karlsruhe Institute of Technology, Institute of Process Engineering in Life Sciences, Section II: Technical Biology, Engler-Bunte-Ring 1, 76131, Karlsruhe, Germany

**Keywords:** *Alternaria alternata*, Mycotoxins, pH value, C:N ratio, Submerged cultivation

## Abstract

Production of the *Alternaria* mycotoxins alternariol (AOH), alternariol monomethylether (AME) and tenuazonic acid (TA) by *Alternaria alternata* DSM 12633 was influenced by pH and carbon to nitrogen (C:N) ratio of the growth medium both in shaking flasks and bioreactor cultivation. The impact of medium pH on mycotoxin production was studied in the range of pH 3.5 - 8. pH values above 5.5 led to a decreased mycotoxin production or inhibited mycotoxin formation completely whereas an acidic pH in the range of 4.0-4.5 was optimal for mycotoxin production. The influence of the C:N ratio was evaluated over the range of 24 to 96. Glucose was used as carbon source and its concentration was altered while nitrogen concentration was kept constant. Growth kinetics and mycotoxin production parameters were studied depending on different C:N ratios. With increasing initial glucose concentration fungal biomass did increase but the maximum specific growth rate was not influenced. The optimal initial C:N ratio for attaining highest mycotoxin concentrations was 72. A higher C:N ratio did not further enhance mycotoxin production.

## Introduction

**Figure 1:**
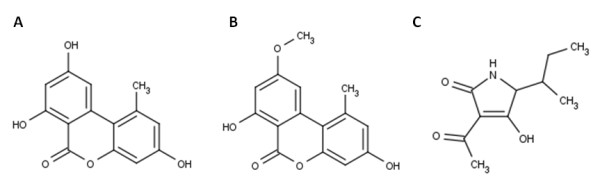
**Chemical structure of the best studied*Alternaria*mycotoxins: alternariol (A), alternariol monomethylether (B) and tenuazonic acid (C).**

Mycotoxins are harmful secondary metabolites produced by a wide variety of moulds. Black moulds of the genus *Alternaria* are able to form several mycotoxins of different chemical classes whereas the polyketide mycotoxins alternariol (AOH) and its methylated derivative alternariol monomethylether (AME) as well as the tetramic acid tenuazonic acid (TA) are best studied ([Coombe et al. 1970]; [Logrieco et al. 2009]; [Patriarca et al. 2007], Figure 1). These compounds have been identified in food products contaminated with *Alternaria* species: wheat ([Li and Yoshizawa, 2000]) and other grains ([Broggi et al. 2007]), sunflower seeds ([Nawaz et al. 1997]; [Pozzi et al. 2005]), oilseed rape ([Nawaz et al. 1997]), pecans ([Schroeder and Cole 1976]), fruit and fruit juices ([Delgado et al. 1996]; [Lau et al. 2003]), tomato products ([Andersen and Frisvad 2004]; [Motta and Valente Soares 2001]; [Ozcelik et al. 1990]) and olives ([Visconti et al. 1986]).

While the acute toxicity of *Alternaria* toxins is low compared to other mycotoxins as e.g. aflatoxins, low-level exposure over long terms can be a serious problem. AOH posses an oncogenic potential and was connected to an incidence of oesophageal cancer in Lixian, China ([Dong et al. 1987]). Although *Alternaria* toxins received increased attention over the last years, e.g. the multidisciplinary project “Safe organic vegetables and vegetable products by reducing risk factors and sources of fungal contaminants throughout the production chain: the carrot - *Alternaria* model” carried out by the European Union from 2000 to 2004, a risk assessment based on the current data is still not possible. In addition to the insufficient toxicological data not much is known about biosynthesis and regulation of the mycotoxins either. Hypothetical biosynthesis pathways for AOH/AME and TA have been suggested ([Gatenbeck and Hermodsson 1965]; [Gatenbeck and Sierankiewicz 1973]; [Hiltunen and Söderhäll 1992]; [Light 1970]; [Stinson 1985]) but the genes are not known. Environmental factors have a great impact on *Alternaria* toxin production. Mycotoxin production varies with fungal strain, substrate and other growth conditions. While TA production seems to be connected to fungal growth ([Brzonkalik et al. 2011a]), production of AOH and AME is not coupled with fungal growth and starts in the late exponential phase ([Söderhäll et al. 1978]). As shown by ([Magan and Lacey 1984]), ([Oviedo et al. 2010]) ([and Pose et al. 2010]) temperature and water activity are important factors influencing AOH/AME and TA production in *Alternaria alternata*. Furthermore light exerts a great influence on AOH production and can reduce mycotoxin formation significantly compared to yields obtained in the dark ([Häggblom and Niehaus 1986]; [Häggblom and Unestam 1979]; [Söderhäll et al. 1978]). ([Brzonkalik et al. 2011b]) studied the influence of different carbon and nitrogen sources in a semi-synthetic medium in static and shaking culture on mycotoxin production in *A. alternata*. They revealed that AOH and AME production is regulated by nitrogen and starts after total nitrogen consumption. The influence of the nitrogen source is higher than of the carbon source. Choice of cultivation condition, carbon and nitrogen source can change mycotoxin composition and determines mycotoxin amount. As shown for different mycotoxins the pH of the medium has an important influence on the expression of the respective biosynthesis genes; In the case of ochratoxin the genes responsible for mycotoxin formation in *Penicillium verrucosum* are highly expressed at pH 8 ([Schmidt-Heydt et al. 2008]) whereas aflatoxin production in *Aspergillus* spp. is increased at acidic pH values of the growth medium ([Keller et al. 1997]). The effect of pH on *Alternaria* toxin production has not been studied yet.

The best way to avoid health risks due to ingestion of contaminated food is the prevention of food spoilage by *Alternaria* toxins. Detailed information of influencing factors and regulatory mechanisms may additionally be helpful for enhanced production of mycotoxins for further toxicological studies. Therefore, profound knowledge about mycotoxin formation is absolutely essential. A robust and highly reproducible platform process in a bioreactor system was developed recently ([Brzonkalik et al. 2011a]) which enables easily the investigation of the influence of different process parameters on *Alternaria* mycotoxin production. The present paper reports on mycotoxin production of *A. alternata* DSM 12633 at different pH values and carbon:nitrogen (C:N) ratios. The effect of these factors on biomass formation was additionally observed in shaking flasks.

## Materials and methods

### Organism and medium

*A. alternata* DSM 12633 was obtained from the DSMZ culture collection (“Deutsche Sammlung von Mikroorganismen und Zellkulturen”, Braunschweig, Germany) and was routinely grown on Potato-Dextrose-Agar (PDA) (Roth, Germany). Conidia were harvested with 25% glycerol from plates that were incubated for 7 days at 28°C and filtered through Miracloth (Calbiochem). Conidia were counted and diluted to 10^6^ conidia per ml. Aliquots were stored at −80°C.

For all experiments modified Czapek-Dox medium (modified after [Gatenbeck and Hermodsson 1965]) was used: 0.06 g/L NH_4_Cl, 0.25 g/L NaNO_3_, 1 g/L KH_2_PO_4_, 0.5 g/L MgSO_4_ x 7 H_2_O, 0.25 g/L NaCl, 0.25 g/L KCl, 0.01 g/L FeSO_4_ x 7 H_2_O, 0.01 g/L ZnSO_4_ x 7 H_2_O, 1 g/L yeast extract. The used yeast extract contained 11% (w/w) nitrogen according to the manufacturer (Becton, Dickinson and Company). The pH was adjusted to 5.5 for the C:N ratio experiments and between pH 3.5 and 8.0 for the pH experiments as indicated. Glucose was added separately after autoclaving. The final concentration in the pH experiments was 10 g/L. For the C:N ratio experiments glucose was added in final concentrations of 10 g/L, 20 g/L, 30 g/L and 40 g/L which corresponds to a C:N ratio of approx. 24, 48, 72 and 96, respectively.

### Cultivation

For fermentation experiments in a bioreactor 1.5 L of modified Czapek-Dox medium were used. The process was operated in the small-scale bioreactor (vessel volume 2.0 L) Minifors (Infors, Switzerland) at 28°C in the dark. The medium was inoculated directly with 1*10^6^ conidia, a pre-culture was not used. The bioreactor was equipped with two 6-blade Rushton Turbines; stirring speed was enhanced from 400 rpm to 900 rpm after 48 h. The aeration rate was 0.013 vvm. For pH adjustment 2 M sodium hydroxide and 2 M phosphoric acid were used.

In the shaking flask experiments 100 ml shaking flasks with baffles were filled with 20 ml of the respective medium and inoculated with 1.7 x 10^4^ conidia. Mycotoxin production, biomass formation, glucose and nitrogen consumption were observed over 7 (pH experiments) or 14 days (C:N ratio experiments). Shaking flasks were incubated at 28°C on a rotary shaker at 140 rpm in the dark. Samples were taken in triplicates. For each sample an individual flask was prepared which was harvested completely.

### Analytical methods

#### Biomass dry weight, glucose and nitrogen concentration

At a given sampling time fungal mycelium was completely removed from the shaking flask and transferred to a pre-weight tube. Biomass was dried in an oven at 60°C and its weight was determined on a standard balance. In bioreactor cultivation experiments biomass was determined at the end of the fermentation.

Glucose concentration was monitored with the photometrical anthrone assay ([Pons et al. 1981]).

For the determination of ammonium and nitrate the photometrical assays “Ammonium-Test” (Spectroquant®, Merck, Germany) and “Nitrat-Test” (Spectroquant®, Merck, Germany) were used according to the manufacturer.

### Mycotoxins

Mycotoxins were extracted and analyzed as described previously ([Brzonkalik et al. 2011a,b]) Briefly, a 10 ml aliquot of cell-free culture broth was acidified with 10 μl conc. HCl (32%, 10.32 M) and extracted twice with 10 ml ethyl acetate. At each extraction step the mixture was vortexed vigorously and centrifuged at 4,600 g for 5 min. The ethyl acetate supernatants from both extraction steps were combined and evaporated to dryness in a vacuum centrifuge. The residue was redissolved in 200 μl methanol (HPLC grade) and used for HPLC analyses. Standards of AOH, AME and TA were purchased from Sigma-Aldrich (Germany).

Mycotoxin standard solutions of 0.2 mM (AOH), 0.1 mM (AME) and 10 mM (TA) in methanol were diluted with methanol and used for calibration. The analysis was performed with a standard HPLC device (Agilent 1100 Series, Agilent, Germany) equipped with a 25 cm reversed phase column (Luna 5 μm C18(2), Phenomenex, Germany) at 30°C. Mobile phase solution was methanol/0.1 M NaH_2_PO_4_ pH 3.2 (2:1) at a flow rate of 0.7 ml/min (according to [Shephard et al. 1991]). Mycotoxins were monitored with a UV detector at 280 nm. Retention times were 5.3 ± 0.1 min for TA, 10.2 ± 0.1 min for AOH and 23.3 ±0.1 min for AME.

To quantify the mycotoxin concentration in the culture broth the peak area in each sample was plotted against the standard curve.

### Data analysis

Glucose consumption and biomass production were fitted using a logistic equation with four parameters in a scientific data analysis and graphing software (Sigma Plot 9.0, Systat, San Jose, USA). The used equation was:

(1) $$y(x)=y_{0}+\frac{a}{1+(\frac{x}{x_{0}})b}$$

The four parameters are the following: y_0_ indicates the minimum concentration of the glucose or biomass; a indicates the maximum glucose/biomass concentration; x_0_ indicates the process time when half of the glucose amount is consumed or half of the maximum biomass concentration is produced; b is a shape parameter and difficult to explain biologically ([Erkmen and Alben 2002]). Derivation of the fitting was used for the determination of absolute consumption and production rates.

## Results

### The influence of pH on mycotoxin production

To elucidate the effect of pH on biomass formation and mycotoxin production experiments were performed in shaking flasks and in a bioreactor. In shaking flasks pH cannot be controlled and it varies over cultivation time but biomass formation can be monitored because each sample consists of a completely harvested shaking flask. In contrast to this, experiments in a bioreactor are highly reproducible with very low standard deviations between single experiments but biomass formation cannot be monitored due to inhomogeneous dispersion of fungal mycelium in the culture broth. The pH was kept constant in bioreactor fermentations due to titration with NaOH and H_3_PO_4_.

**Figure 2:**
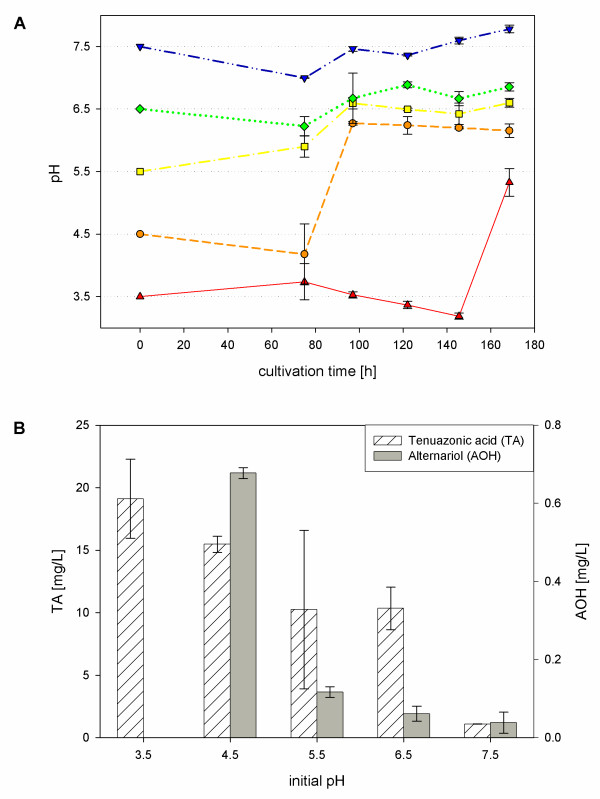
**pH variation (A) and mycotoxin production (B) during shaking flask cultivation of*A. alternata*DSM 12633 in modified Czapek-Dox medium depending on initial pH.** Data is given as an average of 3 independent samples.

In shaking flask experiments a pH range between 3.5 and 7.5 was studied. Figure 2 shows variation of pH during cultivation time and maximum produced mycotoxin concentrations. The pH varied considerably up to 2 pH units between day 3 and 4 of cultivation. During this time highest increase in biomass was observed. In all experiments nearly the same total biodrymass of approx. 5.3 g/L was reached. At an initial pH of 3.5 the course of pH and accordingly biomass increase were delayed compared to the other initial pH values. A clear raise in biomass formation was observed from 120 h cultivation time. At the end of cultivation at 168 h growth was not fully completed. At this initial pH fungal growth was possible after a pH change by the fungus. In the bioreactor conidia did not germinate at pH 3.5, therefore pH 4.0 was tested additionally. In shaking flask experiments detected mycotoxin concentrations raised with dropping initial pH-values. Lowest TA and AOH concentrations were observed at pH 7.5 (1.087 ± 0.014 mg/L TA, 0.038 ± 0.027 mg/L AOH), highest TA concentration were detected at pH 3.5 (19.121 ± 3.159 mg/L), whereas a maximum AOH concentration was measured at pH 4.5 (0.677 ± 0.014 mg/L). AOH was not detected at pH 3.5, AME could not be observed in any shaking flask experiment. In bioreactor experiments the same pH values were tested as in shaking flask experiments. As mentioned above, instead of pH 3.5 pH 4.0 was tested because fungal conidia did not germinate at pH 3.5. The pH was kept constant in bioreactor experiments. The total mycotoxin concentrations were higher as in shaking flasks but less biomass was formed. AME production was observed during fermentation. As in shaking flask experiments highest mycotoxin concentrations were produced at acidic pH values (Table 1). Highest AOH concentrations (5.7 mg/L) were detected at pH 4.0 which corresponds to an increase of 63% compared to the platform process at pH 5.5. A considerable increase in TA production was observed at pH 4.5: TA concentration rose to 54.58 mg/L which corresponds to a 43% enhancement. Highest AME concentrations were also detected at pH 4.5, but the production was only slightly enhanced to 1.73 mg/L. At pH values higher than 5.5 mycotoxin production was significantly reduced. At pH 6.5 AOH (0.73 mg/L) and TA (16.28 mg/L) were detected, at pH 7.5 only AOH formation was observed (0.61 mg/L) and at pH 8.0 mycotoxin production was totally inhibited. As mentioned above, biodrymass concentrations were in all cases lower than in the corresponding shaking flask experiments; approx. 3.3-3.5 g/L biodrymass were measured, at pH 4.0 and 4.5 biodrymass was slightly enhanced up to 4.43 g/L.

**Table 1:** **Mycotoxin production of*A. alternata*in a bioreactor depending on pH and C:N ratio**

**Glucose**	**pH**	**max. AOH**	**max. AME**	**max. TA**	**DW end**	**Reference**
**[g/L]**		**[mg/L]**	**[mg/L]**	**[mg/L]**	**[g/L]**	
10	4.0	5.70	1.09	42.52	4.43	This study
10	4.5	4.39	1.73	54.58	3.76	This study
10	5.5	3.49 ± 0.121	1.62 ± 0.142	38.28 ± 1.61	3.30	[Brzonkalik et al. 2011a]
10	6.5	0.73	n.d.	16.28	3.40	This study
10	7.5	0.61	n.d.	n.d.	3.55	This study
10	8.0	n.d.	n.d.	n.d.	3.30	This study
30	5.5	11.47	6.82	44.26	5.47	This study

DW: dry weight of biomass; n.d.: not detected.

**Figure 3:**
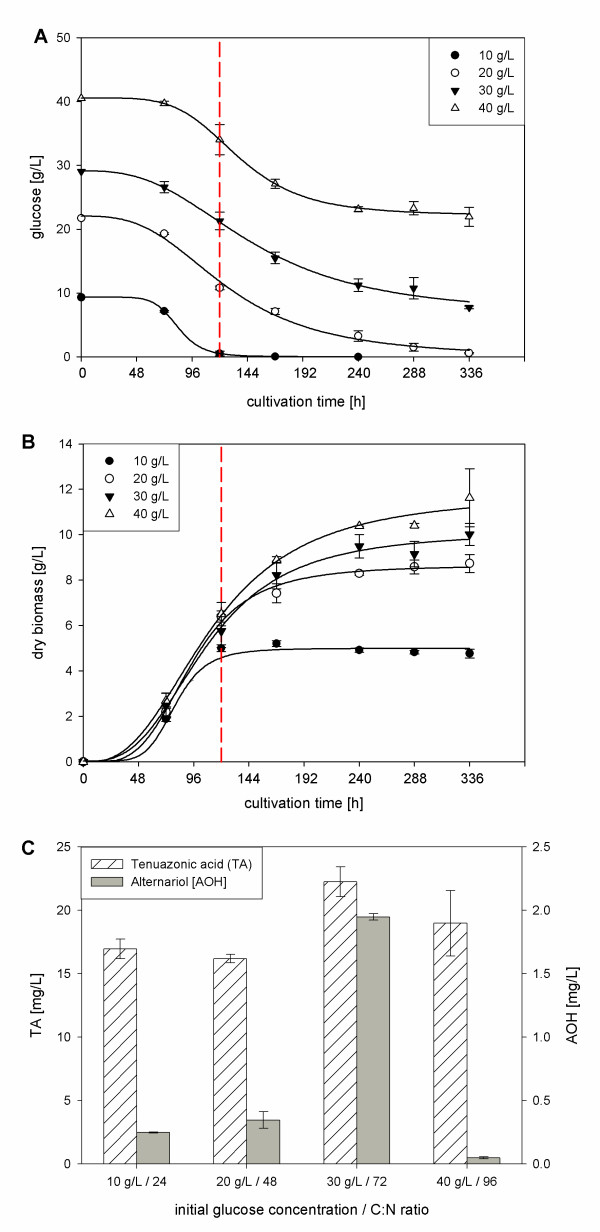
**Glucose consumption (A), biomass production (B) and mycotoxin formation (C) during shaking flask cultivation of*A. alternata*DSM 12633 in modified Czapek-Dox medium depending on C:N ratio.** Data is given as an average of 3 independent samples.

The influence of carbon:nitrogen (C:N) ratio on mycotoxin and biomass formation. Although total mycotoxin concentration and biomass formation in shaking flask experiments are lower than in bioreactor cultivations, they are very suitable to determine tendencies of both parameters and are therefore used for a first screening. To elucidate the influence of the C:N ratio on mycotoxin formation in *A. alternata* 4 different glucose concentrations at constant nitrogen concentrations (167.8 mg/L) were tested in shaking flask experiments: 10 g/L, 20 g/L, 30 g/L and 40 g/L which corresponds to a C:N ratio of 24, 48, 72 and 96, respectively. Figure 3 shows glucose consumption, biomass formation and maximum mycotoxin concentration at the respective C:N ratios. The red dashed line in Figure 3A and 3B represents the total exhaustion of nitrogen. In all experiments nitrogen was the limiting nutrient and was consumed after 5 days (data not shown). Glucose consumption and biomass formation can be sufficiently fitted by a logistic equation with 4 parameters (1). With an initial glucose concentration of 10 g/L glucose was completely consumed after 5 days.

Therefore, biomass increased only until day 7 to 5.2 g/L and kept constant from then on. It can be assumed that glucose given at an initial concentration of 10 g/L was also a limiting factor in these experiments. In all other experiments independent on initial glucose concentration approximately 20 g/L glucose were consumed over the observed 14 days and glucose consumption curves were quite similar. According to Figure 3B the logistic growth phase can be located between day 3 and 5. After nitrogen consumption at day 7 the stationary growth phase was reached and only slight increases in biomass were observed. Highest biomass yields (11.62 g/L) were achieved with initial glucose concentration of 40 g/L after 14 days. Table 2 summarizes the effect of initial glucose concentration on fungal growth (by maximum dry weight). Independent on the glucose concentration the maximum specific growth rate μ_max_ and the maximum growth rate are almost the same in all experiments and were calculated to be 0.029 - 0.045 h^-1^ and 0.079 - 0.095 g/h, respectively. As for the growth kinetics mycotoxin production was observed depending on different C:N ratios. Figure 3C shows maximum TA and AOH concentrations. AOH was initially detected at day 5 when nitrogen was depleted. A maximum AOH concentration can be observed at day 7. For the initial glucose concentrations of 10 g/L, 20 g/L and 40 g/L nearly the same AOH concentrations were detected: 0.25 mg/L ± 0.003, 0.12 mgL/± 0.07 and 0.33 mg/L ± 0.29, respectively. AOH production was noticeably enhanced at an initial glucose concentration of 30 g/L, 1.95 mg/L ± 0.03 AOH could be achieved. TA was formed in the early growth phase as it was already detected at day 3. TA concentration courses followed biomass increase. The maximum TA concentrations were 16.95 mg/L ± 0.78, 16.18 mg/L ± 0.34, 22.25 mg/L ± 1.17 and 18.97 mg/L ± 2.58 at initial glucose concentrations of 10 g/L, 20 g/L, 30 g/L and 40 g/L, respectively. As with AOH production the initial glucose concentration of 30 g/L seemed to favor TA production, but the enhancement appeared not to be significant and may be explained by higher biomass production. Additionally, cultivation with 30 g/L glucose was performed in the bioreactor. Compared to the platform process with 10 g/L glucose ([Brzonkalik et al. 2011a]) AOH and AME production was enhanced considerably to 11.47 mg/L and 6.82 mg/L (Table 1) which corresponds to a 3-fold and 4-fold increase, respectively. As in the shaking flask experiments TA production was only slightly enhanced to 44.26 mg/L.

**Table 2:** **Effects of initial glucose concentrations/different C:N ratios on fungal growth of* A. alternata***

**Parameter**	**Initial glucose concentration [g/L] (C:N ratio)**			
	**10 (24)**	**20 (48)**	**30 (72)**	**40 (96)**
**max. DW**^**a**^**[g/L]**	5.20 ± 0.13	8.73 ± 0.39	10.02 ± 0.48	11.62 ±1.28
**μ**_**max**_**[h**^**-1**^**]**^**b**^	0.045	0.039	0.032	0.029
**max. growth rate r**_**x**_**[g/h]**^**b**^	0.095	0.093	0.079	0.082
**Average growth rate [g/d]**	0.74	0.62	0.72	0.83
**Y**_**X/S**_**[g/g]**	0.52	0.44	0.45	0.64

All results refer to day 14 excepting the results for 10 g/L glucose which refer to day 7. ^a^ Results are mean of three replicates ± standard deviation.

^b^ obtained by deviation of Eq. 1.

DW: dry weight of biomass.

## Discussion

Although the influences of temperature and water activity on the production of *Alternaria* toxins were studied extensively, the influence of other abiotic factors was neglected so far. In this study the effects of the two important factors, pH-value and C:N ratio, were elucidated for the first time and it was shown that mycotoxin production was highly affected by these factors.

In general, the regulation of most of fungal toxins is very complex and one special regulator does not exist. As shown by many studies before, pH value exerts a great influence on the production of different mycotoxins. Although the optimal pH value has to be determined individually for each mycotoxin, the production of most of the mycotoxins is increased at acidic pH values. ([Keller et al. 1997]) reported highest sterigmatocystin production in *Aspergillus nidulans* and highest aflatoxin production in *A. parasiticus* at pH 4.0. By buffering ammonium growth media in a range from pH 4 to 8, they observed an approximately 5-fold decrease in mycotoxin production in both *Aspergillus* spp. with increasing pH. According to the detected mycotoxin concentrations they observed that the transcript levels of the aflatoxin/sterigmatocystin pathway genes *ver-1* and *stc-U* first appeared at acidic pH values and only later or not at all in neutral or alkali conditions. Similarly, much higher levels of ochratoxin are produced by *A. ochraceus* in the lower pH range, with a 4-fold reduction in levels being observed when the pH of the growth medium was increased from pH 4 to pH 10 ([O'Callaghan et al. 2006]). Likewise, a low pH regulates deoxynivalenol production in *Fusarium graminearum* ([Gardiner et al. 2009]). ([Gardiner et al. 2009]) observed that a decrease of the initial pH from pH 4.0 to 3.5 led to a 2-fold increase in deoxynivalenol concentration. They also showed that an initial pH between 2.4 and 3.1 was required to induce deoxynivalenol production when *F. graminearum* was previously grown in non-producing medium.

In agreement with our shaking flask results they reported a rapid acidification of the growth medium due to ammonium consumption ([Morton and Macmillan 1954]; [Jernejc and Legiša 2001]) followed by a increase to pH 8. They suggested that the drop in pH prior to the start of deoxynivalenol biosynthesis may be required to release a possible inhibition by the global pH regulatory system mainly mediated by the transcription factor PacC. PacC homologues play a role in the regulation of many mycotoxins, e.g. fumonisin production of *F. verticillioides* ([Flaherty et al. 2003]) and sterigmacystin biosynthesis in *A. nidulans* ([Delgado-Virgen and Guzman-de-Peña 2009]). Consequently, it is possible that alternariol production is regulated by a PacC homologue as well. Applying a blast search of PacC of *A. nidulans* (GenBank: CAA67063.1) against the sequenced and annotated genome of the close relative *Alternaria brassicicola* (http://genome.jgi-psf.org/Altbr1/Altbr1.home.html) revealed that *A. brassicicola* harbors a PacC homologue as well (jgi|Altbr1|4090|AB04090.1, E-Value: 3.25E-97). To clarify whether alternariol biosynthesis is regulated by a PacC homologue the identification of the biosynthesis cluster is necessary. Nevertheless, our results from the bioreactor studies showed that in contrast to ([Gardiner et al. 2009]) a drop in pH is not necessary to induce alternariol production because the pH was kept constant in the bioreactor. Furthermore, alternariol production in shaking flasks started after the end of growth phase when the pH of the medium was already increased. So, in *A. alternata* mycotoxin production is induced by low pH values, but a change in pH is not necessary.

With respect to the C:N ratio experiments it was already shown before that this ratio is important for the production of secondary metabolites. ([Casas Lopéz et al. 2003]) studied the influence of different C:N ratios on lovastatin production. They found that the presence of excess carbon under nitrogen limitation greatly enhanced the rate of production of lovastatin and determined an optimal C:N ratio of ~40. They stated that lovastatin production is associated with the nitrogen limited stationary growth phase when excess carbon can be channeled into secondary metabolism. The same can be applied to alternariol production. [Brzonkalik et al. (2011a]) showed clearly the secondary metabolite character of alternariol which is produced in the stationary growth phase after nitrogen depletion. An excess of carbon has a positive impact on alternariol production but does not affect growth rate or yield. In contrast to alternariol tenuazonic acid is formed growth associated and contains nitrogen. Therefore, an increase in carbon concentration does not alter TA production. These results clearly emphasize the different regulatory mechanisms for both mycotoxins and were supported by the results of the bioreactor cultivation.

## Competing interests

The authors declare that they have no competing interests.
